# Electrical, Mechanical and Electromechanical Properties of Graphene-Thermoset Polymer Composites Produced Using Acetone-DMF Solvents

**DOI:** 10.3390/polym10010082

**Published:** 2018-01-16

**Authors:** Alessandro Giuseppe D’Aloia, Alessandro Proietti, Hossein Cheraghi Bidsorkhi, Alessio Tamburrano, Giovanni De Bellis, Fabrizio Marra, Agnese Bregnocchi, Maria Sabrina Sarto

**Affiliations:** 1 Department of Astronautical, Electrical and Energy Engineering, Sapienza University of Rome, via Eudossiana 18, 00184 Rome, Italy; alessandro.proietti@uniroma1.it (A.P.); hossein.cheraghibidsorkhi@uniroma1.it (H.C.B.); alessio.tamburrano@uniroma1.it (A.T.); giovanni.debellis@uniroma1.it (G.D.B.); fabrizio.marra@uniroma1.it (F.M.); agnese.bregnocchi@uniroma1.it (A.B.); mariasabrina.sarto@uniroma1.it (M.S.S.); 2 Research Center on Nanotechnology Applied to Engineering of Sapienza (CNIS), Sapienza University of Rome, via Eudossiana 18, 00184 Rome, Italy

**Keywords:** graphene-polymer composites, acetone-DMF solvents, electromechanical properties

## Abstract

Recently, graphene-polymer composites gained a central role in advanced stress and strain sensing. A fundamental step in the production of epoxy-composites filled with graphene nanoplatelets (GNPs) consists in the exfoliation and dispersion of expanded graphite in a proper solvent, in the mixing of the resulting GNP suspension with the polymer matrix, and in the final removal of the solvent from the composite before curing through evaporation. The effects of traces of residual solvent on polymer curing process are usually overlooked, even if it has been found that even a small amount of residual solvent can affect the mechanical properties of the final composite. In this paper, we show that residual traces of *N*,*N*′-Dimethylformamide (DMF) in vinylester epoxy composites can induce relevant variations of the electrical, mechanical and electromechanical properties of the cured GNP-composite. To this purpose, a complete analysis of the morphological and structural characteristics of the composite samples produced using different solvent mixtures (combining acetone and DMF) is performed. Moreover, electrical, mechanical and electromechanical properties of the produced composites are assessed. In particular, the effect on the piezoresistive response of the use of DMF in the solvent mixture is analyzed using an experimental strain dependent percolation law to fit the measured electromechanical data. It is shown that the composites realized using a higher amount of DMF are characterized by a higher electrical conductivity and by a strong reduction of Young’s Modulus.

## 1. Introduction

Over the last decade, graphene-polymer composites have attracted growing attention for both civil and military applications [1,2]. In fact, the development of metal-free conductive fillers has driven the interest for electrically conductive polymer composites which can combine good electrical properties with the typical characteristics of polymeric materials, such as lightweight, high formability and resistance against corrosion [2,3]. Generally, the electrical conduction in a polymer composite depends on the formation of conductive fillers networks throughout the insulating polymer matrix [4,5,6,7]. Nevertheless, high filler loadings can lead to a decrease in mechanical strength and ductility as well as to poor processability. Within this context, nanofillers have attracted growing attention from the scientific community. Amongst the nanofillers, two-dimensional graphene nanoplatelets (GNPs) have monopolized the recent literature. GNPs are carbon nanostructures consisting of small stacks of graphene sheets, with thicknesses typically in the range of 1–10 nm and lateral linear dimensions much greater, varying from about 1 μm up to 20–25 μm [8,9]. GNP based polymer composites have been widely investigated in various engineering applications, including electromagnetic compatibility [9,10,11,12,13], protection from electrostatic discharge (ESD) [14,15], structural sensing and monitoring [16,17,18,19,20,21].

Nowadays, in the production process of such polymer composites a solvent is often used to exfoliate expanded graphite through ultrasonication, thus facilitating the dispersion of GNPs into the polymer matrix. Excess solvent is removed from the composite by evaporation before curing. Nevertheless, residual solvent traces may remain trapped within the composite and consequently they can affect the functional properties of the resulting material. However, the effect of solvent residues is usually overlooked by the scientific community, which is more focused on the effect of the filler itself on the final properties of the composite, rather than the effects on the curing process [22,23]. Indeed, even a small amount of residual solvent has been found to be sufficient to markedly affect the mechanical properties of the final composite. For instance, Mondragon and Bucknall investigated the curing process of neat epoxy resins with or without dichloromethane as solvent, proving that even a small amount of this chemical measurably influences the cure kinetics and glass transition temperature *T*_g_ of the final resin, affecting the mechanical properties [24].

A solvent commonly used for the production of carbon based polymer composites is *N*,*N*′-Dimethylformamide (DMF) [25]. To date, its effect on the final carbon based composite is not well defined, but it can be envisioned that trapped DMF at high temperature may decompose into amine [22]. The as-generated amine could act as a terminating agent, reacting with epoxide group and altering the network structure. The final result is a decrease of crosslink density, leading to a deterioration of mechanical properties. However, the use of DMF as solvent may increase the electrical conductivity of GNP based composites, since GNP exfoliation and dispersion in DMF is more efficient, as demonstrated in [26,27]. As a consequence, DMF may be used for the production of electrically conductive GNP based polymer composites, but its action during curing and its effect on the mechanical properties of the composite should be carefully taken into account.

Scope of this paper is to investigate the electrical, mechanical and electromechanical properties of graphene-polymer composites prepared through exfoliation of expanded graphite in a solvent mixture containing acetone and DMF. To this purpose, GNP-filled composites were produced using different mixtures, consisting of various ratios of acetone and DMF, as solvent for expanded graphite exfoliation and GNP uniform dispersion, and a commercial epoxy-based vinyl ester resin as polymer. The produced composites were characterized from both morphological and chemical points of view, and their electrical, mechanical and electromechanical properties were investigated experimentally. The effect on the piezoresistive response of the use of DMF in the solvent mixture was analyzed using an experimental strain dependent percolation law to fit the measured electromechanical data. It is shown that the composites realized using a higher amount of DMF show a higher electrical conductivity and a strong reduction in Young’s Modulus.

## 2. Materials and Methods

### 2.1. GNP Production and Composite Fabrication

GNPs were derived upon thermal expansion of Graphite Intercalation Compounds (GICs) [11], provided by Graftech Inc. (GrafTech International Holdings Inc., Brooklyn Heights, OH, USA). According to the producer, the GICs mean lateral size is 350 µm. Before obtaining GNPs, an intermediate step, involving a thermal shock, had to be performed. To this purpose, GNPs were placed in a muffle furnace heated at 1150 °C, for 5 s. This step caused a GIC’s volume increase of up to 400 times, leading to the formation of thermally expanded graphite oxide (TEGO). After the thermal treatment, TEGOs were dispersed in a suitable solvent, and the obtained suspension was tip sonicated using an ultrasonic processor, thus obtaining GNPs [12]. A good control of the GNPs morphology was achieved constraining TEGO concentration in the solvent in the range 1.5–3 mg/mL, and using beakers with the same capacity for all preparations.

Polymer composites were prepared by using an epoxy-based vinyl ester product (DION 9102 produced by Reichhold (Durham, NC, USA)). This resin has an initial viscosity of 150–200 MPa·s, a density of 1.01–1.05 g/cm^3^, and a styrene content around 50 wt %.

Upon completion of the sonication step, the GNP suspension and the vinyl ester resin, previously activated with 0.2 wt % of a co-based accelerator, were mixed together and further sonicated for 30 s using a low ultrasound amplitude, with the aim of minimizing bubble formation. 

Next, the mixture was magnetically stirred at 200–250 rpm, in order to remove the solvent in excess. Upon complete evaporation of the solvent, the hardener was added at 2 wt % ratio.

**Figure 1:**
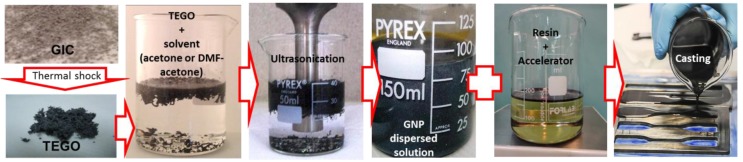
Graphene nanoplatelet (GNP) composite fabrication process.

The resulting mixture was finally stirred again at 250 rpm, until it gained a viscosity suitable for casting, and then it was poured directly in to a 3 mm-thick dog-bone shaped brass mold, in order to produce specimens suitable for tensile tests, according to [28]. Then, the produced composite specimens were cured in air for 24 h and post-cured for another 24 h at 70 °C, and finally polished and extracted from the molds. The fabrication process is sketched in Figure 1.

The characteristics of the produced samples are summarized in Table 1. The samples were named using: the letter A to indicate that only acetone has been used as solvent; the letters AD to indicate that the samples were produced using a mixture of 90 vol % acetone and 10 vol % DMF as solvent; the letter B to indicate that the solvent mixture used in the exfoliation process is DMF at 3 vol % and acetone at 97 vol %. The number following the letters corresponds to the GNP % weight content with respect to the total resin amount.

### 2.2. Morphological Investigation

Field-emission scanning electron microscopy (FE-SEM) and scanning transmission electron microscopy (STEM) were employed to analyze the morphology of GICs, TEGOs, GNPs and nanocomposite samples, by using a Zeiss Auriga FE-SEM (Oberkochen, Germania) available at SNN-Lab of Sapienza University of Rome. Prior to the microscopic analysis, nanocomposite specimens were broken in liquid nitrogen and sputter coated with a 10 nm Cr layer, by using a sputter coater (Quorum Tech Q150T, Quorum Technologies Ltd., Laughton, UK).

Further characterization of GNP flakes was carried out through Atomic Force Microscopy (AFM), using a Bruker Dimension Icon AFM (Bruker, Billerica, MA, USA), operated in tapping mode. In order to realize specimens suitable for AFM imaging, 10 μL aliquots of a GNP-acetone suspension, were drop cast onto a 300 nm SiO_2_ coated Si wafer chip and the resulting samples were allowed to dry at 100 °C for 10 min in a laboratory oven.

### 2.3. Fourier Transform Infrared Spectrometer

Fourier transform infrared (FTIR) spectroscopy has been carried out at ENEA, Frascati Research Center, in order to investigate possible variations in the polymer structure due to polymer-solvent interactions (either with acetone or with acetone-DMF mixtures). 

All the FTIR absorption spectra were recorded using a FTIR spectrometer (Tensor 27 by Bruker Optics GmbH, Bruker, Billerica, MA, USA) equipped with a single reflection diamond attenuated total reflectance (ATR) cell. The analyses were performed into the typical mid infrared (IR) (Bruker, Billerica, MA, USA) spectral range (1800–500 cm^−1^) at a resolution of 1 cm^−1^ and with 20 scans per minute.

### 2.4. Dynamic Mechanical Thermal Analysis

Dynamic Mechanical Thermal Analysis (DMA) was performed at the Institute of Polymer Science and Technology, Madrid, Spain, using a Mettler DMA861e (Mettler Toledo, Columbus, OH, USA). The Storage Modulus *E*′ and the tanδ of each sample were measured as a function of temperature *T*. The first attribute describes the ability of the material to store elastic energy and to immediately release it upon removal of the related stress, while tanδ is related to the dissipation of energy. Furthermore, the glass transition temperature *T*_g_ is evaluated as the value of the abscissa corresponding to the peak of the tanδ vs. temperature curve [29].

Each sample was tested at different frequencies (1, 5, 10 and 30 Hz), sweeping the temperature between 25 to 200 °C. The test was carried out by applying a sinusoidal stress to beam-shaped samples (length 19.5 mm, width 5 mm, thickness 1.5 mm). 

### 2.5. Electrical Conductivity Measurement

**Figure 2:**
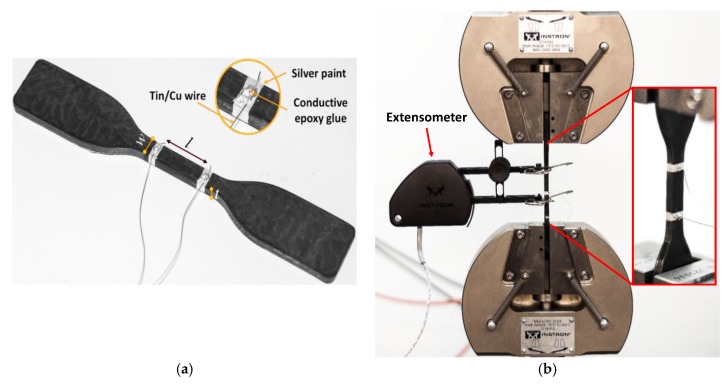
Photographs of the dog-bone sample prepared for dc electrical conductivity measurement (*w* = 6 mm, *l* = 15 mm, *t* = 4 mm) (**a**) and of the test setup used for electromechanical characterizations (**b**). The GNP-based specimen is placed between the grips of an Instron 3366 universal testing machine.

The effective dc electrical conductivity γ_eff_ of the produced nanocomposites was obtained using the following equation:

(1) $$\gamma_{\mathrm{eff}}=l{\left(wt\;R\right)}^{-1}$$

where, as shown in Figure 2a, *w* and *t* are the width and thickness of the gage section of the dog-bone shaped specimen, *l* and *R* are the distance and measured resistance between the two-ring electrical contacts, deposited on the material surface using a silver-based paint (Electrolube^®^, Ashby de la Zouch, UK).

The resistance *R* of the samples loaded with GNPs at 1, 1.5 and 2 wt % was measured using a dc/ac current source (Keithley 6221, Keithley Instruments, Cleveland, OH, USA) and a nano-voltmeter (Keithley 2182A, Keithley Instruments, Cleveland, OH, USA). On the other hand, the resistance of samples having a lower GNP content was measured using an Electrometer/High Resistance Meter (Keithley 6517B, Keithley Instruments, Cleveland, OH, USA). Two tin-coated copper wires, properly bonded to the silver contacts through a conducting epoxy glue (Circuitworks^®^, Chemtronics, Kennesaw, GA, USA), were used to connect the samples to the measuring set-up (Figure 2a).

It is worth noticing that the electrical measurements were performed in a controlled environment (temperature of 23 ± 0.5 °C and humidity of 35 ± 5%), and all the samples, before testing, were dried for 24 h in a desiccator.

### 2.6. Mechanical and Electromechanical Tensile Tests

An Instron 3366 universal testing machine equipped with a 10 kN load cell (Dept. of Astronautical, Electrical and Energy Engineering of Sapienza, Roma, Italy) was used to perform mechanical tensile tests of the produced samples, according to [28]. The geometry of the tensile specimens is reported in Figure 2a; the surfaces were accurately polished before testing in order to minimize the flaw influence on the measured mechanical properties.

The electromechanical properties of the considered GNP-based nanocomposites were then analyzed by measuring the variation of the dc electrical resistance as a function of the applied tensile strain. An extensometer was used for strain measurement and the crosshead speed was set at 0.5 mm/min. The experimental setup is shown in Figure 2b; the electrical resistance was measured using the setup described in Section 2.4.

## 3. Results

### 3.1. Morphology

**Figure 3:**
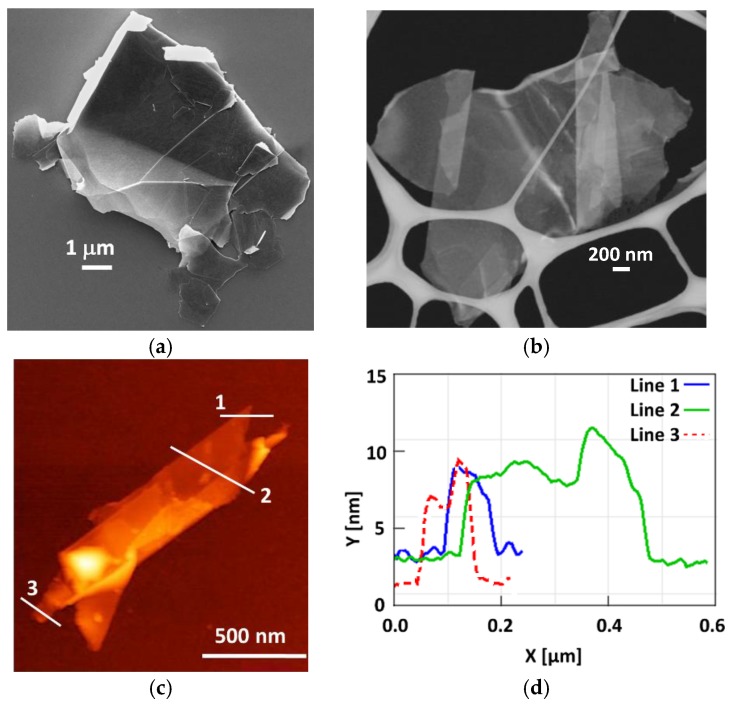
GNP flakes morphology characterizations: (**a**) SEM and (**b**) STEM micrographs, (**c**) AFM image and (**d**) profiles of lines 1, 2 and 3.

Figure 3a,b show respectively the SEM and STEM micrographs of two GNP flakes. It appears that GNPs have lateral dimensions in the micrometer-range (between 1 and 10 μm), they are characterized by an irregular geometry and by sharp edges and wedges. Moreover, from AFM characterizations, it results that the produced GNPs have an average thickness varying from ~4 up to ~13 nm (Figure 3c,d).

**Figure 4:**
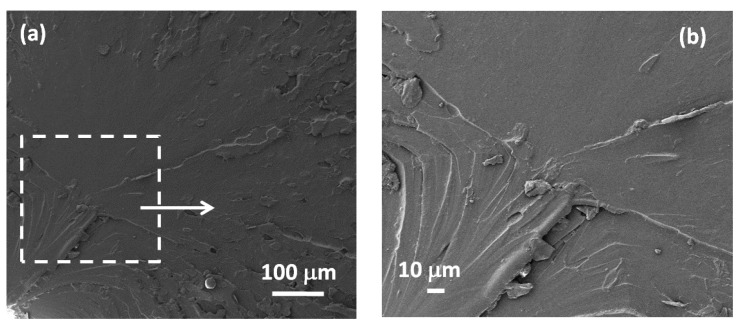

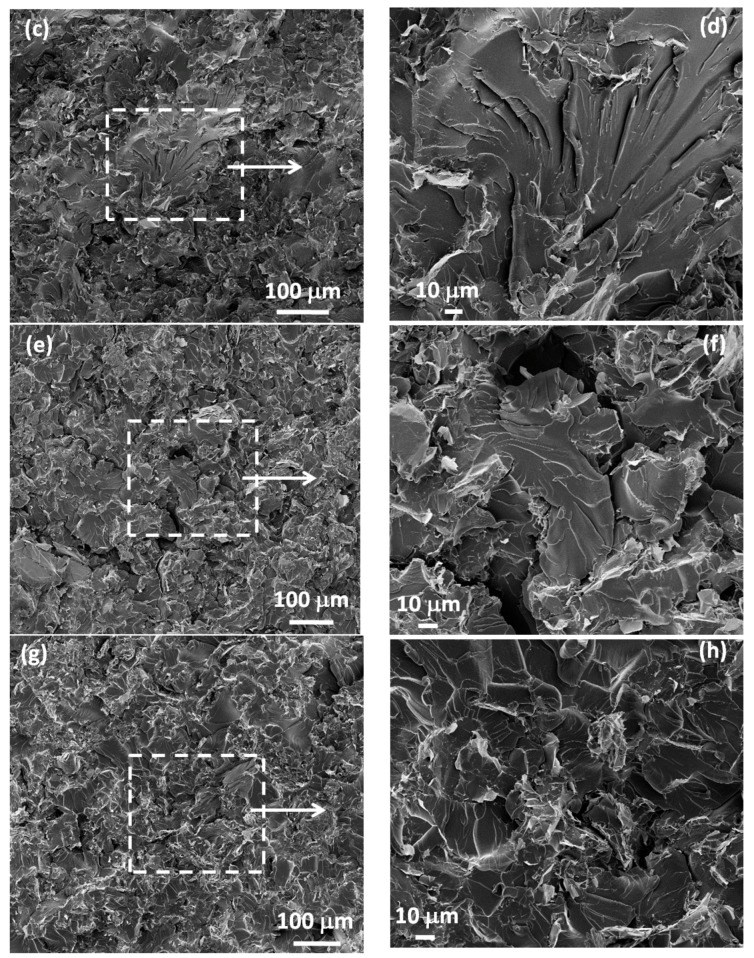
SEM micrographs of the cross-section of neat resin specimen (**a**,**b**) and of samples of composite filled with 1 wt % GNPs and prepared using different solvent mixtures: (**c**,**d**) sample A-1.0 prepared with only acetone as solvent; (**e**,**f**) sample AD-1.0 prepared using a mixture of 90% acetone and 10% DMF as solvent; (**g**,**h**) sample B-1.0 prepared using a solvent mixture of 97% acetone and 3% DMF.

Figure 4 shows the SEM images of the fracture surface of the produced specimen of neat resin (a), (b) and of the different types of composites filled at 1 wt % GNPs (c)–(h), in order to reveal the effect of the use of different solvents during processing.

It appears that the surface of the neat polymer sample (Figure 4a,b) is rather smooth and it does not show cracks or defects. Moreover, we notice that in all of the composite samples, GNPs are dispersed quite uniformly without formation of agglomerations. Nevertheless, the fracture surfaces of the composite samples show some cracks and defects, which become relevant in samples produced using acetone-DMF mixtures as solvent (samples AD-1.0 and B-1.0 in Figure 4e–h). These cracks are particularly evident in the samples produced using the highest amount of DMF in the mixture (i.e., sample AD-1.0 in Figure 4e,f). In fact, it is observed that the fracture surfaces of samples AD-1.0 and B-1.0 are characterized by larger fractures and cracks than the ones of sample A-1.0 (Figure 4c,d), prepared using only acetone as solvent. This is probably due to the decrease of cross-links in the vinylester matrix caused by the use of DMF as discussed in [22]. In particular, the surface of sample AD-1.0 (Figure 4e,f) is characterized by larger fractures (up to several microns in width and a few tens of microns in length) than the surface of sample B-1.0 (Figure 4g,h), prepared with a lower content of DMF (Table 1). In fact, fractures observed in Figure 4g,h have dimensions up to 1–2 microns in width and up to ten microns in length.

### 3.2. Fourier Transform Infrared Spectrometer

The IR spectrum of the samples produced using different solvent mixture was acquired in the spectral range between 1800 and 500 cm^−1^ with the aim of analyzing the interactions between the polymer and the solvents.

**Figure 5:**
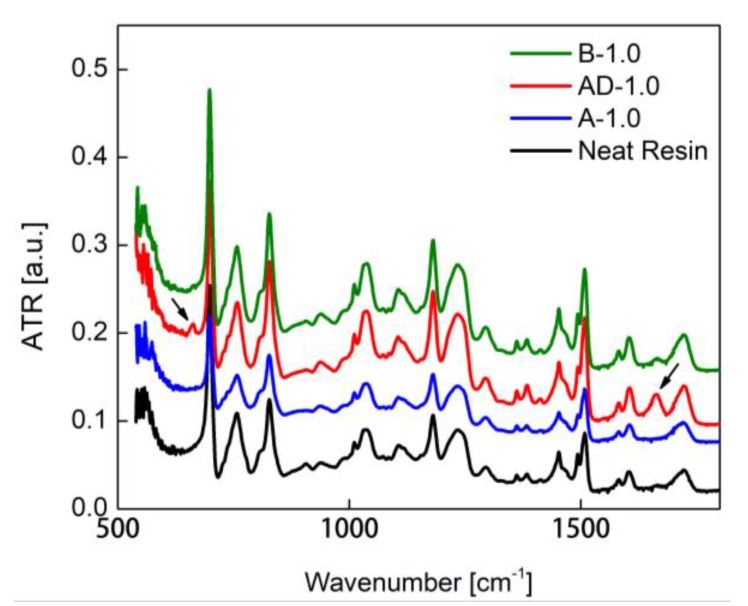
FTIR absorption spectra of neat resin and of samples A-1.0, AD-1.0 and B-1.0, with the two additional peaks originated by the presence of DMF in the solvent mixture used for processing.

Figure 5 shows the FTIR absorption spectra of a neat resin specimen and of samples A-1.0, AD-1.0 and B-1.0, all filled at 1 wt % of GNP, but produced using different solvents, as reported in Table 1. It can be noticed the emergence of two new peaks when DMF is used in the solvent mixture (i.e., for samples AD-1.0 and B-1.0). These peaks are located at 661 and 1662 cm^−1^, and are generally associated to O–C=O and C=O chemical groups. In particular, the 661 cm^−1^ peak can be attributed to the hydrogen bonding between polymer hydrogen atoms and DMF carbonyl group oxygen atoms. However, the peak at 1662 cm^−1^ is referred to dimethylamine, an aliphatic secondary amine generated during the curing process due to DMF decomposition. The so generated secondary amine may react with the resin epoxy groups, acting as a terminator rather then a co-hardener, which changes the network structure and decreases the crosslink density [22,30,31,32,33,34]. Finally, the FTIR spectra of A-1.0 sample (produced using only acetone) does not show any extra peak. Thus, we conclude that acetone does not react with polymer chemical groups.

### 3.3. Dynamic Mechanical Thermal Analysis

**Figure 6:**
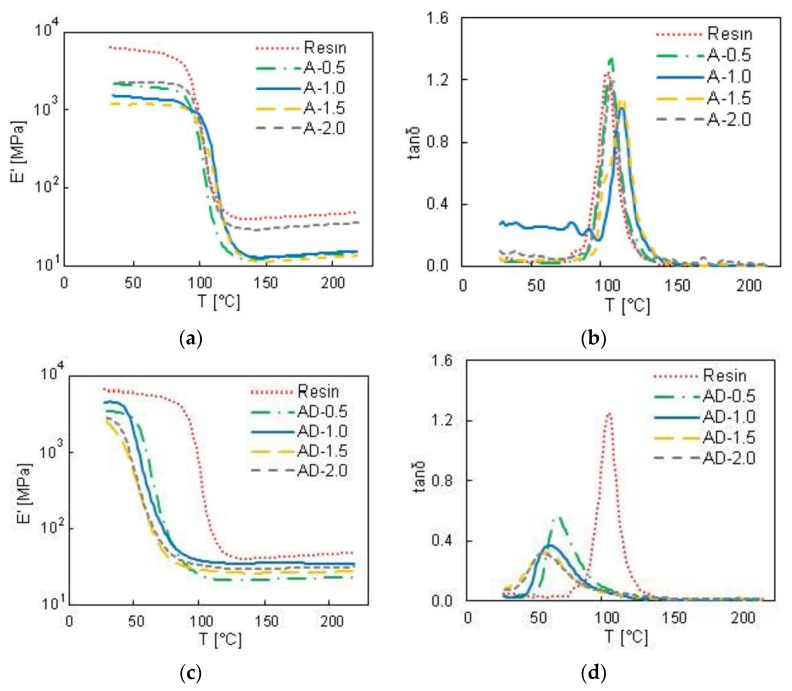
Storage modulus *E*′ (**a**,**c**) and tanδ (**b**,**d**) values as a function of the temperature for nanocomposites with GNP content from 0.5 to 2 wt %, obtained using only acetone as solvent (**a**,**b**) and 90% acetone, 10% DMF in the solvent mixture (**c**,**d**).

DMA analysis were performed at 1 Hz on the samples A-0.5, A-1.0, A-1.5, A-2.0 and AD-0.5, AD-1.0, AD-1.5, AD-2.0, characterized by a GNP concentration varying from 0.5 to 2 wt % and by the use of acetone or a mixture of 90 vol % acetone and 10 vol % DMF as solvent. The measured values of *E*′ and tanδ are reported in Figure 6, as functions of the temperature *T*. A completely different behavior of the sample produced using pure acetone (Figure 6a,b) with respect to the ones produced using the acetone-DMF mixture (Figure 6c,d) is observed.

From Figure 6a it can be noticed that the GNP filled samples produced using only acetone as solvent show lower storage modulus than the neat resin. Furthermore, from the tanδ plot of Figure 6b, it can be observed that the glass transition temperature of the GNP filled samples produced using only acetone increases slightly compared to the neat resin one, from ~111 up to ~125 °C for sample A-2.0. This rise can be attributed to the intrinsic crystalline effect of graphene on polymer composites, i.e., to a restriction in molecular motion and to a higher degree of crosslinking, indicating significant changes in polymer chains dynamics [35].

**Figure 7:**
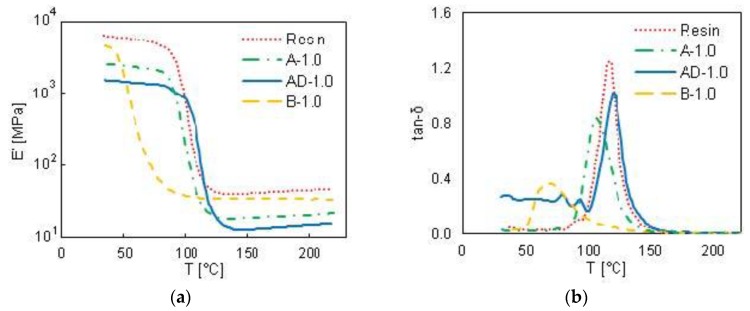
Storage modulus *E*′ (**a**) and tanδ (**b**) values as a function of the temperature of samples A-1.0, AD-1.0 and B-1.0, all filled with 1 wt % of GNPs.

However, from the comparison of Figure 6a,c and Figure 7a,b, it is clear that the samples produced using a mixture of acetone and DMF as solvent have lower storage modulus in comparison with neat resin and samples fabricated using only acetone. In addition, it is noticed that the glass transition temperature decreases strongly (around 38%) when the solvent mixture includes DMF. This can be attributed to the lower crosslinking density and to the higher mobility of the polymer chains due to the presence of DMF. In fact, the residual DMF changes the cured matrix structure from a homogeneous and highly crosslinked network to a heterogeneous broadly-distributed crosslinked structure with lower crosslinks density [22]. This effect is confirmed also by the presence of crack and defects observed through SEM imaging in samples produced using DMF, as shown in the previous section.

### 3.4. Mechanical Properties

**Figure 8:**
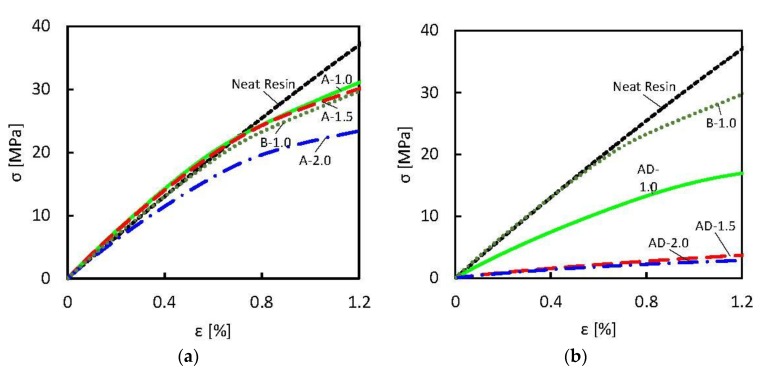
Measured tensile stress-strain characteristic of GNP-filled composites, produced using: only acetone (**a**) or acetone and DMF (**b**) as solvent for GNP exfoliation. For sake of comparison, the mechanical responses of neat resin and sample B-1.0 with the lowest content of DMF are reported in both the graphs.

The results of the tensile test performed on samples A-1.0, A-1.5, A-2.0 and AD-1.0, AD-1.5, AD-2.0 are shown in Figure 8a,b, respectively. These results are compared with the ones obtained for the neat resin and for sample B-1.0, produced using a lower amount of DMF. Then, the extrapolated Young’s Modulus is reported in Table 2.

It is noticed that the A-type samples (without DMF in the solvent mixture) are characterized by a Young’s Modulus higher than the one of the neat resin for GNP concentration up to 1.5 wt %. On the contrary, the composites produced using acetone-DMF mixture as solvent are characterized by a Young’s Modulus lower than the one of the neat resin for all GNP concentration. In particular, it is observed that samples prepared using a mixture of 90 vol % acetone and 10 vol % DMF as solvent are characterized by a strong reduction of Young’s Modulus, whereas for a minimum content of DMF (i.e., sample B-1.0) this reduction is within 10–15% of the value measured for the neat resin.

It is also observed that the maximum stress recorded during the tensile test decreases for all samples, and this reduction is more evident for the composites produced using DMF in the solvent mixture.

The reduction of the mechanical properties can be addressed mainly to two different factors: for filler concentration higher than 1–1.5 wt %, the formation of GNP agglomerates induces the formation of mechanical defects in the composite; for composites produced using DMF in the solvent mixture, the presence of DMF deteriorates the mechanical properties of the resin, as pointed out in Section 3.2 and Section 3.3 and discussed in [22,36,37,38]. For these reasons, composites produced using a larger amount of DMF in the solvent mixture show an evident reduction of the maximum stress and of the Young’s Modulus.

As a confirmation, sample B-1.0, obtained using a lower amount of DMF, shows similar mechanical strength and properties with respect to sample A-1.0, obtained without the use of DMF.

### 3.5. Electrical Properties

**Figure 9:**
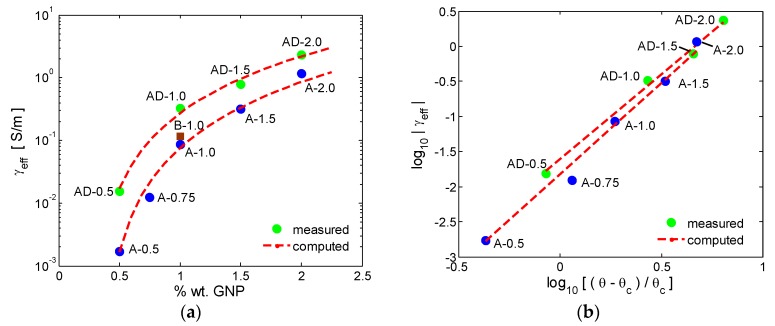
DC conductivity of GNP-filled composite samples produced using acetone and acetone-DMF mixtures, as function of the filler weight concentrations (**a**) and least-squares fit of log10(γ_eff_) versus log10[(θ − θ_c_)/θ_c_] for the same composites (**b**).

The measured effective dc conductivity γ_eff_ of the produced GNP based composites is reported in Figure 9a, as a function of the percent filler weight fraction θ. As the GNP concentration increases, the graphene-polymer composites undergo an insulator-to-metal transition. The basic reason for this transition is the presence of conducting fillers inside the polymeric matrix. Below a critical concentration of conducting fillers (i.e., the percolation threshold θ_c_), conduction throughout the material cannot take place. At the percolation threshold, a rapid increase in the material electrical conductivity takes place, since the GNPs form a network of connected paths through the insulating polymer matrix [39].

Above the percolation threshold, the composite effective conductivity γ_eff_ scales with the filler concentration θ, according to the well-known percolation law [8,39,40]:

(2) $$\gamma_{eff}\left(\theta \right)=K{\left(\theta -\theta_{c}\right)}^{t}$$

where *t* is the critical exponent and *K* a determination coefficient, dependent on the material characteristics and processing. According to the classical percolation theory, the value of *t* depends only on the dimensionality of the percolating system [39,40].

**Figure 10:**
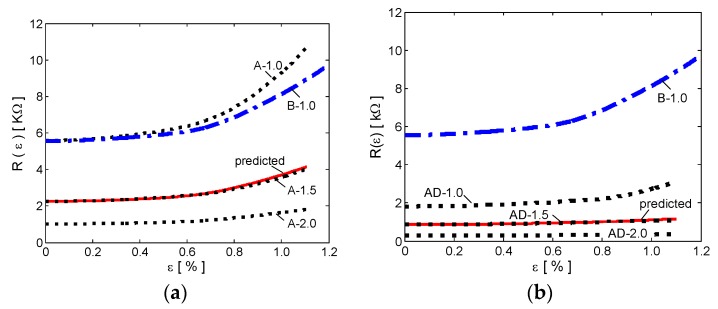

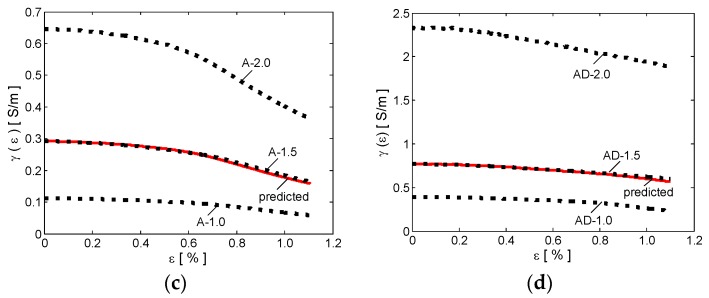
Resistance change as a function of strain for nanocomposites with GNP content of 1, 1.5 and 2 wt %, obtained starting from a GNP-acetone (**a**) and a GNP-acetone-DMF (**b**) mixture. Conductivity change as function of strain for the same nanocomposites obtained starting from a GNP-acetone (**c**) and a GNP-acetone-DMF (**d**) mixture.

The values of *t*, θ_c_, and *K* for the composites of type A or AD are estimated from the best fit of the measured value of electrical conductivity of the produced samples with increasing GNP content (from 0.5 to 2 wt %) with the percolation law given in (3). For the A-type composites (i.e., samples A-0.5, A-0.75, A-1.0, A-1.5, A-2.0) the best fit is obtained setting θ_c_ = 0.35 wt %, *t* = 2.6, and *K* = 36,754 S/m. For the AD-type composites (i.e., samples AD-0.5, AD-1.0, AD-1.5, AD-2.0), it results θ_c_ = 0.27 wt %, *t* = 2.408, and *K* = 38,183 S/m. The straight line in Figure 10b represents the least-squares fit of log10(γ_eff_) versus log10[(θ − θ_c_)/θ_c_].

It is noticed that samples produced using an acetone-DMF mixture as solvent are characterized by a higher conductivity in comparison with the samples realized without the use of DMF. This is due to the higher mobility of the polymer chains due to the presence of DMF and to the better dispersion of GNPs in DMF than in acetone. Such increase in the electrical conductivity of the composites is observed even for very low amounts of DMF, as observed in sample B-1.0.

### 3.6. Electromechanical Response

The electromechanical response of the produced samples with a minimum GNP concentration of 1 wt % has been characterized by measuring the electrical resistance as a function of the applied strain, as described in Section 2.5. The samples with a GNP content of 0.5 wt % have not been characterized, since the measured resistance value was too high for sensing applications.

The measured electrical resistance of the produced samples is shown in Figure 10a,b (dotted lines). The corresponding effective dc conductivities have been extracted from the measured values of the sample resistances using Equation (1) and neglecting the variations of the specimen geometrical dimensions. The results are shown in Figure 10c,d.

The obtained curves are nearly constant in the linear elastic range (i.e., for induced strain up to ~0.5%). For higher strain values, we observe a rapid increase of the electrical resistance with *ε*. This reveals a strong piezoresistive response of the produced samples. According to [39,40], this piezoresistive phenomenon can be mainly attributed to the propagation of small cracks in the composite and to the degradation of the GNP conducting network inside the polymer. In fact, a loss of contact between adjacent fillers and a change of the tunneling resistance in neighboring GNPs take place, modifying the final composite conductivity.

**Figure 11:**
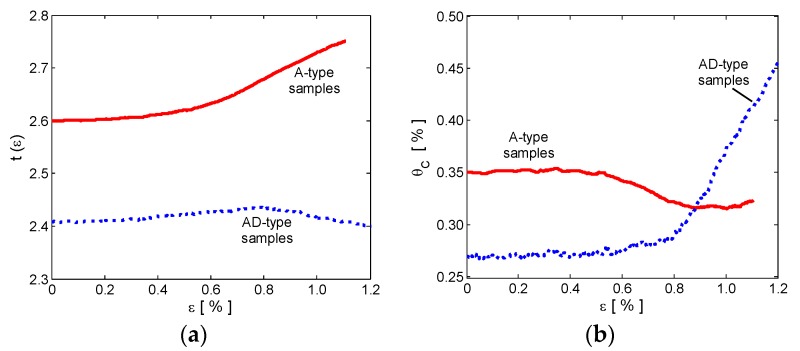
Estimated strain dependent percolation threshold (**a**) and critical exponent (**b**) for A-type (solid line) and AD-type (dotted line) samples.

In order to better understand the effect of DMF on the piezoresistive response of the produced samples, the measured data were fitted with a strain dependent percolation law, obtained considering the percolation formula (2) in which the percolation threshold θ_c_ and the critical exponent *t* are assumed to be strain-dependent quantities. On the contrary, the determination coefficient *K* is assumed to be constant since it depends only on the material characteristics and processing route. Therefore, Equation (2) becomes:

(3) $$\gamma_{\mathrm{eff}}\left(\varepsilon \right)=K{\left[\theta -{\tilde{\theta }}_{c}\left(\varepsilon \right)\right]}^{\tilde{t}\left(\varepsilon \right)}$$

where ${\tilde{\theta }}_{c}\left(\varepsilon \right)$ and $\tilde{t}\left(\varepsilon \right)$ are the strain dependent percolation threshold and critical exponent, respectively. These functions are computed numerically for each composite type (i.e., the A-type and the AD-type) through the solution of the non-linear system obtained by the substitution of γ_eff_(#x3B5;) in Equation (3) with the values of electrical conductivity measured as a function of the strain for the samples filled with 1 and 2 wt % of GNPs. The obtained strain-dependent functions ${\tilde{\theta }}_{c}\left(\varepsilon \right)$ and $\tilde{t}\left(\varepsilon \right)$ for the A-type and the AD-type composites are reported in Figure 11a,b.

In order to validate the model, the calculated strain-dependent percolation threshold and critical exponent functions were used to predict the electromechanical response γ_eff_(ε) of the two samples filled at 1.5 wt % (i.e., A-1.5 and AD-1.5). It is noticed that the experimental data are almost perfectly overlapping the computed results, for both A-1.5 and AD-1.5 samples. Thus, we conclude that the strain dependent ${\tilde{\theta }}_{c}\left(\varepsilon \right)$ and $\tilde{t}\left(\varepsilon \right)$ are representative of the electromechanical properties of the produced composite materials.

Looking at Figure 11a,b, it is evident that in the linear elastic region (i.e., for ε up to ~0.5%), ${\tilde{\theta }}_{c}\left(\varepsilon \right)$ and $\tilde{t}\left(\varepsilon \right)$ for both composite types are nearly constant with strain. As the applied strain increases, the percolation threshold of A-type samples decreases slightly, while the critical exponent increases. A totally different behavior is observed in the AD-type composites, in which the critical exponent decreases slightly, while the percolation threshold increases rapidly beyond 0.8% strain.

The main reason for this totally different behavior is that the use of DMF as solvent changes significantly the composite structure and properties, as discussed above (Section 3.1, Section 3.3, Section 3.4 and Section 3.5). In fact, the secondary amine generated during the curing process due to DMF decomposition, reacts with the resin epoxy groups and acts as a terminator, thus decreasing the crosslink density of the composite. The resulting DMF-processed samples are characterized by a heterogeneous and broadly-distributed structure with larger cracks than the ones realized without the use of DMF (Figure 4a–h). As a consequence, AD-type samples are characterized by a higher crack density under stress than the A-type ones. When a strain is applied, the resulting stress focuses on cracks and produces an increase of the inter-particle distance, which corresponds to an increase of the percolation threshold θ_c_. Accordingly, the critical exponent *t* tends to decrease since transition from non-conducting to conducting regime becomes smoother. On the contrary, in DMF-free composites the higher crosslinks density guarantees a better distribution of the stress inside the composite. This results in a stretching of the polymeric chains inside the composite and in a faster transition from the non-conducting to the conducting regime in the nonlinear region.

## 4. Conclusions

The electrical, mechanical and electromechanical properties of graphene-polymer composites produced using different acetone and acetone-DMF mixtures as solvents were investigated. The produced GNP based composites were morphologically and chemically characterized, and the electrical, mechanical and electromechanical properties were tested. All the experimental results show that DMF clearly affects the electrical and mechanical properties of the composites. In fact, samples produced using a higher amount of DMF in the solvent mixture are characterized by a higher electrical conductivity with respect to the corresponding ones produced without the use of DMF. On the other hand, DMF clearly affects the mechanical properties, since all the composites produced using DMF show an evident decrement of the maximum stress and of the Young’s modulus. As a confirmation of DMF effect on the final electrical and mechanical properties, sample B-1.0, produced using only 3 vol % of DMF in the solvent mixture, show similar mechanical and electrical properties of the corresponding sample realized without the use of DMF. These effects are originated by the lower crosslink density of DMF-processed composites, which are characterized by a higher defectiveness.

Moreover, an experimental strain dependent percolation law, relating the composite electrical resistance with the applied tensile strain is used to investigate the effect on the piezoresistive response of the use of DMF in the solvent mixture. It is demonstrated that composite produced through a DMF-free process are characterized by an almost constant percolation threshold with respect to strain but by an increasing critical exponent, which is representative of the strain-induced stretching of the polymeric chains and results in a faster transition from the non-conducting to the conducting regime. 

Conversely, in the DMF-processed composites, the piezoelectric response fits well a strain-dependent percolation curve characterized by an increasing percolation threshold and an almost constant critical exponent for strain higher than ~0.5%. This is representative of the presence of DMF-induced cracks and defects in the polymer matrix, and results in a gelification of the composite, as discussed also in the recent literature [22,30,31,32,33,34].

## Figures and Tables

**Table 1:** List of the produced composite specimen, including GNP wt % concentration and composition of the solvent mixture used for GNP exfoliation.

Sample name	GNP (wt %) *	Solvent	
		Acetone (vol %) **	DMF (vol %) **
A-0.5	0.50	100	0
A-0.75	0.75	100	0
A-1.0	1.00	100	0
A-1.5	1.50	100	0
A-2.0	2.00	100	0
AD-0.5	0.50	90	10
AD-1.0	1.00	90	10
AD-1.5	1.50	90	10
AD-2.0	2.00	90	10
B-1.0	1.0	97	3

* wt % concentration expressed with respect to the total resin weight. ** vol % concentration expressed with respect to the total volume of the solvent mixture.

**Table 2:** Tensile elastic modulus of GNP-based composites with GNP concentration ranging between 1 and 2 wt %.

Sample	Young’s modulus (GPa)
Neat resin	3.25 ± 0.03
A-1.0	3.54 ± 0.04
A-1.5	3.50 ± 0.06
A-2.0	2.87 ± 0.14
AD-1.0	1.87 ± 0.09
AD-1.5	0.47 ± 0.15
AD-2.0	0.31 ± 0.11
B-1.0	3.20 ± 0.03
